# Microglial Function in the Effects of Early-Life Stress on Brain and Behavioral Development

**DOI:** 10.3390/jcm9020468

**Published:** 2020-02-08

**Authors:** Clarissa Catale, Stephen Gironda, Luisa Lo Iacono, Valeria Carola

**Affiliations:** 1 Department of Psychology, University of Rome “Sapienza”, Via dei Marsi, 78, 00185 Rome, Italy; clarissa.catale@gmail.com (C.C.); luisa.loiacono@yahoo.it (L.L.I.); 2 IRCCS Santa Lucia Foundation, Via Fosso di Fiorano 64, 00143 Rome, Italy; 3 Department of Physiology and Pharmacology, Wake Forest School of Medicine, Winston-Salem, NC 27157, USA; sgirond1@villanova.edu; 4 Department of Dynamic and Clinical Psychology, University of Rome “Sapienza”, Via degli Apuli 1, 00185 Rome, Italy

**Keywords:** microglia, early life stress, prenatal stress, postnatal stress, behavioral stress, environmental agents, infection, immune system, brain development, behavior

## Abstract

The putative effects of early-life stress (ELS) on later behavior and neurobiology have been widely investigated. Recently, microglia have been implicated in mediating some of the effects of ELS on behavior. In this review, findings from preclinical and clinical literature with a specific focus on microglial alterations induced by the exposure to ELS (i.e., exposure to behavioral stressors or environmental agents and infection) are summarized. These studies were utilized to interpret changes in developmental trajectories based on the time at which the stress occurred, as well as the paradigm used. ELS and microglial alterations were found to be associated with a wide array of deficits including cognitive performance, memory, reward processing, and processing of social stimuli. Four general conclusions emerged: (1) ELS interferes with microglial developmental programs, including their proliferation and death and their phagocytic activity; (2) this can affect neuronal and non-neuronal developmental processes, which are dynamic during development and for which microglial activity is instrumental; (3) the effects are extremely dependent on the time point at which the investigation is carried out; and (4) both pre- and postnatal ELS can prime microglial reactivity, indicating a long-lasting alteration, which has been implicated in behavioral abnormalities later in life.

## 1. Introduction

**Figure 1:**
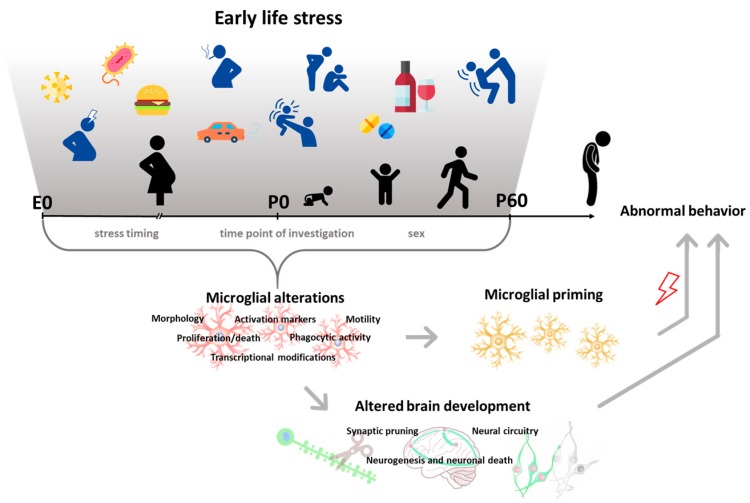
Microglia mediate the effects of early life stress (ELS) on brain and behavioral development. E: embryonic day; P: postnatal day.

Exposure to childhood adversities (i.e., abuse, neglect, physical illness, economic difficulty) is a risk factor for developing psychopathologies later in life. A history of early-life stress (ELS) is strongly associated with almost all classes of mental disorders, including mood disorders, anxiety disorders, and substance use disorders [1]. Biological embedding such as alterations in brain structure, function, connectivity, and epigenetic programming has been consistently reported in individuals exposed to adversities in childhood [2,3]. It has been suggested that exposure to ELS could modify the trajectories of brain maturation [4,5,6], increasing vulnerability to psychopathologies later in life. However, neurobiological mechanisms underlying the emergence of these changes have been poorly characterized. Recently, microglial cells have been proposed as mediators of the neurodevelopmental abnormalities induced by ELS exposure [7]. Microglia are the resident macrophages of the brain, which scan the surrounding extracellular space to maintain homeostasis [8]. If a danger signal is encountered, these cells undergo rapid morphological and functional changes (phenotypic changes) that include the phagocytosis of triggering stimuli and the synthesis and secretion of inflammatory mediators. During development, microglia are extremely active and contribute intensively to shaping and refining the developing neural circuits by regulating neurogenesis, synaptogenesis, synaptic pruning, and ultimately behavior [9,10,11,12]. Changes in microglial function during development have been demonstrated to affect these processes [13,14]. Crucially, accumulating evidence has reported that ELS can perturb microglia and their activity. In this review, we summarize the literature surrounding ELS and microglia to explore the role of these cells in ELS-induced aberrant development (Figure 1). We describe preclinical data showing the impact of pre- and postnatal adversities, in the form of harmful environmental stimuli, on microglia in rodents. Moreover, we report studies providing evidence for altered microglial–neuronal communication and microglia-dependent behavioral alterations following ELS. Finally, we discuss clinical findings in the field.

## 2. Discussion

### 2.1. Early-Life Stress and Microglia: Preclinical Studies

As sensors of the surrounding environment, microglia are highly responsive to local perturbations. These perturbations can drive different reactive states in microglia through morphological and functional changes. Interestingly, environmental stressors other than immune stimuli can trigger a microglial response, and this phenomenon has been proposed to contribute to shaping the organism’s response to threats [15]. Indeed, microglial receptors’ repertoire allows them to sense and respond to signaling from stress-reactive networks, including the endocrine, immune, and nervous systems [15,16].

When occurring in early life, stress can induce different microglial responses that may result in two main consequences. First, interfering with microglial phagocytic activity and neuronal–microglial signaling can disrupt neural circuits’ developmental trajectories and alter the formation of behavior [17]. Second, aberrant functionality of maturing microglial cells can alter their developmental programs, with long-lasting consequences for their reactivity. Indeed, stress exposure in early life can “prime” microglia to be more responsive to subsequent challenges later in life, leaving a permanent memory of the stressful experience with only partially known consequences in adulthood [17,18].

In rodent models, multiple ELS manipulations (i.e., exposure to behavioral stressors or environmental agents and infection) have been shown to induce microglial changes in offspring. In this review, we explore similarities and divergences in preclinical evidence surrounding pre- and postnatal stress effects on microglia, based on the type of ELS procedure employed, the developmental timing with which the stress was introduced, and the time point at which the effects were examined. Specifically, we have categorized stress into two major groups. The first, behavioral stressors, includes paradigms that have been proven to induce activation of the hypothalamic–pituitary–adrenal axis (i.e., increased corticosterone in rodents) or administration of molecules of the stress response pathway (i.e., glucocorticoids) that mimics a stressful condition. The second category, environmental agents and infection, consists of factors such as exposure to poor diet, drugs of abuse, chemicals, and immune activators which have been demonstrated to affect development. Within this categorization, differences between early/short-term effects measured immediately after the stress exposure and late/long-term effects measured later in life are discussed, along with resulting differences in sex-specific outcomes.

Microglial alterations that have been more commonly investigated include cell density changes and immunophenotypic shifts, as reflected by consistent variations in cell morphology and expression of microglial markers. These changes have been mostly detected by ex vivo immunohistochemical analysis, using antibodies that recognize such markers, including ionized calcium-binding adapter molecule (IBA-1) or clusters of differentiation (CD) molecules, such as complement receptor 3 (CD11b/c), CD68, and CD74. Specifically, morphological and transcriptional variations characterize the “activated” microglia, which usually display reduced length, thicker processes, and larger cell somas (hypertrophic/bushy microglia) or no processes (amoeboid), compared to resting microglia, which are small-bodied and highly ramified [16]. Notably, during development, the degree of ramification reflects the maturity of microglial cells, and an amoeboid morphology is thought to reflect an immature phenotype [19,20].

Few studies have examined the transcriptional changes of signaling molecules implicated in microglial–neuronal interactions, such as the fractalkine (CX3CL1)/fractalkine receptor (CX3CR1) system, the CD200/CD200R system, and the P2Y purinergic receptor 12 (P2Y12) [21,22].

To investigate the impact of ELS-induced microglial alterations on brain and behavior development, studies have mostly used minocycline as a pharmacological tool with which to prevent or counteract microglial activation. Despite its low specificity (effects on other immune cells, subtle effects on astroglial activation) and controversial mechanism of action (possible effects on microbiota), minocycline has been primarily used in the past—and still is—thanks to its favorable kinetic properties and its relatively low cost [23,24,25]. Very few studies have applied methodologies for analysis of the specific contribution of microglia/macrophages, rather than other cells, in these processes. These techniques include, for example, new pharmacological tools that reversibly ablate microglia, such as inhibitors of colony-stimulating factor 1 receptor (CSF1R; e.g., GW2580).

Finally, only one clinical study to date has specifically analyzed microglia in individuals who have been exposed to ELS. This study employed the positron emission tomography (PET) radioligand [11C]PBR28, which binds to the 18 kDa mitochondrial translocator protein (TSPO), which is thought to be expressed in active microglia during brain injury and neurodegeneration and is considered a marker of neuroinflammation [26,27].

#### 2.1.1. Prenatal Behavioral Stressors

The earliest evidence suggesting a relationship between prenatal stress and microglia was posited in 2010 by Gómez-González and Escobar. In their study, pregnant rats were exposed to a *forced swimming task* throughout gestation. The exposure to stress via the dam induced a brain-region-specific effect on microglia of postnatal day (P)1 offspring. An overall decrease in microglial cell number and a reduction in amoeboid microglia were observed in the corpus callosum. Instead, an increase in the number of microglial cells (mainly ramified) was detected in the entorhinal and parietal cortices, the septum, amygdala (AMY), and thalamus [28].

Since Gómez-González and Escobar (2010), few studies have considered the effects of prenatal behavioral stress on microglial activation. Studies conducted by Zhao et al. (2014, 2015) investigated the effects of *maternal sleep deprivation* (three-hour deprivation starting at embryonic day, E, 18 until birth) on pup microglia at P21. This manipulation resulted in a 30% increase in microglial density and retracted thicker processes in the hippocampus (HIP) [29,30].

Consistent with these results, *maternal restraint stress* (from E12–P21) in female mice produced a 40% increase in microglia, larger somas, and shortened branching in the pup HIP [31].

#### 2.1.2. Prenatal Environmental Agents and Infection

The *maternal immune activation* (MIA) model [32] has been used to explore the immediate and late effects of prenatal stress on microglia [33]. Interestingly, microglia have been implicated in the vulnerability of MIA offspring to developing neuroimmune and behavioral abnormalities, especially after exposure to a second stressful challenge later in life [34,35,36,37,38,39]. Evidence suggests that poly (I:C) injections (a commonly used MIA model) at E9 increase amoeboid microglial cells in both sexes at P62 [36]. However, a sex-dependent long-term sensitivity to this stress was demonstrated in more recent studies, as this phenotype was still visible at P90 only in males in the HIP [40]. Moreover, the expression of CX3CR1 in the HIP was only reduced in P15 males exposed to lipopolysaccharide (LPS, another MIA model) injection at E9 [39]. These findings generally support a common effect of MIA on microglia. However, research suggests that there may be critical periods of development during gestation in which microglia are more sensitive to dysregulation induced by MIA. For example, minocycline treatment in adulthood has been shown to rescue alterations in microglia activation after poly (I:C) injections at E9 but not E15 [34,36]. Moreover, LPS injection at E13.5 is not able to increase microglia cell numbers in the somatosensory cortex of P7 mice [40].

In contrast to the studies above, Giovanoli et al. (2013) found that poly (I:C) injection (E9) alone was not sufficient to induce microglial activation (P56). However, when this procedure was combined with peripubertal variable stress, an activated microglial phenotype was observed in the HIP and prefrontal cortex (PFC) at P41 through increased expression of specific markers and soma enlargement [41]. Minocycline during peripubertal stress exposure was found to ameliorate the effects of the combined stress on microglia activation [37].

Consistent with these findings, injection of poly (I:C) at E15 enhances microglial activation in the HIP and increases cell density in the corpus callosum at P180 [35].

Diet is a critical determinant of neonatal brain development, and a poor diet is the most common cause of immunodeficiency [42]. A Western *high-fat diet* (HFD) consisting of 40% fat provided to the dam throughout gestation until P21, in conjunction with limited nesting material, induced significant pro-inflammatory alterations to microglia in the HIP of both sexes at P21, including increases in cell number and in IBA-1 and toll-like receptor (TLR) 9 expression [43]. In a study by Edlow et al. (2019), however, an analogous HFD regimen similarly altered microglia cells in the HIP only in male but not female rats at E17 [44].

A combination of perinatal brain injury induced by a gestational *low-protein diet* and postnatal *injections of interleukin-1β* (IL-1β), caused an upregulation of inflammation-related gene sets and an increase in microglial cell number in cortical white matter at P4. Cultured microglial cells from P2 low-protein-diet animals displayed a more amoeboid morphology and released higher concentrations of several pro-inflammatory cytokines with respect to controls [45].

Prenatal exposure to *alcohol* induces significant alterations to microglia activity [46]. Evidence suggests that prenatal alcohol through maternal exposure during gestation causes an increase in microglial cell number and activation at E15.5 and P3 [46].

Studies have sought to combine prenatal exposure to *diesel exhaust particles* and HFD throughout adolescence to investigate how these environmental factors can influence microglia activity and behavior [47,48]. These studies demonstrated that diesel exhaust particles and HFD increased microglial density, and colocalization of microglia and neurons in the HIP of males while having no effect on females at P63. Although this was consistent with other HFD studies [43,44], further evidence presented by Bolton et al. (2017) showed that diesel exhaust particles were sufficient to induce these changes to microglial activity without a high-fat diet, which may, in part, have been due to a dysregulation in TLR4 signaling [48].

#### 2.1.3. Effects of Prenatal-Stress-Induced Microglial Alterations on Brain and Behavior

Although there has been little investigation on the effect of prenatal stress on microglial alterations and their impact on circuit formation and behavior, several studies have demonstrated effects. Zhao et al. (2014, 2015) demonstrated that *maternal sleep deprivation* increases microglia count, anhedonia-like behavior, impairments to spatial learning and memory, and decreases neurogenesis at P21. In a follow-up study, minocycline was shown to recover deficits in both behavior and neurogenesis, suggesting that maternal sleep deprivation’s effects are microglia-dependent [29,30].

Prenatal *exposure to alcohol* reduces mRNA expression of signal transducers such as CX3CL1, insulin-like growth factor 1, and brain-derived neurotrophic factor (BDNF) in the HIP [46].

Thion and colleagues (2019) recently showed that prenatal *LPS injection* (E13.5) and embryonic microglia depletion (E6.5/E7.5 injection of an antibody that targeted the CSF1R) induced remarkably similar alterations in parvalbuminergic interneuron wiring in the somatosensory barrel cortex, suggesting that MIA effects could result from an inability of microglia to fulfill their standard physiological roles [40]. In addition, a poly (I:C) injection at E15 resulted in an adult-like microglial transcriptome across the brain at P1 [49], while the same injection at E14 caused a reduction in gene expression related to cell activation, immune response, motility, and phagocytosis in adulthood [50]. Regarding behavior, pups prenatally exposed to poly (I:C) showed an alteration in startle response, repetitive behavior, and locomotor activity [34,35,36,37,38,39], which were then rescued by postnatal minocycline treatment [30,34,36,37] (see Table 1 for a summary).

#### 2.1.4. Postnatal Behavioral Stressors

The first studies exploring the effects of postnatal stress on microglia investigated the responses of these cells to *early glucocorticoids* in order to mimic a psychological stress condition. In 1955, Field showed that exposure to the stress hormone cortisone in newborn (P3–5) rats reduced microglial cells [51]. In 1982, Ling confirmed and expanded these results, reporting a 50% reduction in the number of amoeboid (immature) microglia in the corpus callosum two days after the cortisone injection, and a complete absence from the fifth postnatal day onwards. Interestingly, at the same time point, most microglial cells observed displayed a ramified and mature morphology, usually appearing later in life [52].

Similarly to cortisone, early postnatal administration of the synthetic glucocorticoid dexamethasone reduced 40%–60% of the number of amoeboid microglia in the corpus callosum of rats (P4, 7). This was associated with a transient reduction in microglial cell proliferation, an increase in microglial cell death, and acceleration of their maturation, which returned to control levels by P8 [53,54].

More recently, studies investigating the effects of behavioral stress procedures in early life have found a general increase in the number and activation of microglial cells across brain areas. Stressing the mouse dam through the *limitation of nesting and bedding material* in the cage induces microglia changes in the pup [55,56]. Early effects comprise reduced processes complexity in the HIP, suggesting the presence of an amoeboid phenotype and reduced number in the entorhinal cortex [56]. However, the late effects of this paradigm have not been reported if not in combination with another early environmental manipulation (unbalanced fatty acid diet), in which limited bedding induced an increase in microglia in the adult HIP [55]. A series of ex vivo investigations on the early effects of *maternal separation* (MS) on microglia showed a general increase in the proportion of cells with an activated morphology (amoeboid/hypertrophic, with large somas and thick short processes) across central nervous system (CNS) regions such as the HIP [57,58,59] and the medulla [60]. Interestingly, in one of these studies, microglial amoeboid morphology was not associated with an augmented phagocytic activity measured in vitro [58]. The impact of MS on the number of microglial cells is not as clear, with studies reporting an increase [58,61], a decrease [59], or no effect [57] on the number of microglial cells or expression of IBA-1 in the HIP. These differential results can be species-specific (rat vs. mouse) or due to the subregion of the HIP considered. Interestingly, Chocyk and colleagues found a decrease in the number of apoptotic microglia cells (expressing the active caspase-9+ marker and exhibiting the morphological hallmarks of apoptosis) in the substantia nigra and ventral tegmental area of MS rats [62]. This suggests that the higher microglial cell count detected after MS could be primarily due to a reduction in microglial cell death, rather than a proliferation.

Several studies have demonstrated that MS exposure has lasting, likely permanent effects on microglia. In general, adult brains from MS mice do not present differences in the number of microglial cells, but show region-specific modulation in the expression of activation markers and phagocytic activity and motility. Specifically, MS induces long-term increases in the expression of IBA-1 in brain regions such as the PFC [61], the dorsal striatum, the nucleus accumbens, and the CA3 subregion of the HIP [63]. However, a decrease in IBA-1 expression was detected in the adult spinal cord from MS rats [64]. In an in vitro assay, hippocampal microglia from adult MS mice showed an increased phagocytic activity [58]. Capturing microglial cells in vivo, Takatsuru and colleagues (2015) showed that somatosensory stimulation in adulthood induced a significantly higher increase in the number of microglial processes formation (motility) in MS mice vs. controls [65]. This response could influence microglia–synapse interactions and, ultimately, neuronal functionality.

Finally, in order to better understand the molecular changes associated with morphological and densitometric alteration after ELS challenges, Delpech and colleagues profiled the gene expression of hippocampal microglia in MS mice (brief daily separation model) immediately after the stress procedure, revealing perturbation of several genes, including an increased expression of genes involved in cell cycle regulation and apoptosis (e.g., CSF1, CSF3R), microglial activation, and anti-inflammatory function, and a reduced expression of several pro-inflammatory genes. Exposure to brief daily separation also modified several phagocytosis-related genes, and some of these transcriptomic alterations were still present later in life [58].

The exposure to stress during adolescence, primarily in the form of social challenges, has a definite impact on microglial functionality, inducing a transient increase in microglial activity and then a decrease of microglial markers later in life. The study by Gong and collaborators (2018) showed that one day of *brief social isolation* at P14 was sufficient to increase microglial density in the HIP, likely by augmenting the proliferation of these cells. After four days of isolation (P14–17), cell number returned to control levels and microglia showed ongoing apoptotic processes. In adulthood, exposure to a week of brief social isolation (P14–21) induced a reduction in the number of microglial cells in the dentate gyrus (HIP). As a result of these findings, the authors suggested that brief social isolation during the third postnatal week triggers a complex process of activation and subsequent apoptosis of microglial cells, resulting in a significant loss of microglia in adults [66]. Similarly, *social defeat* in adolescent mice provokes an early augmentation in PFC IBA-1 and a subsequent reduction in microglial cells and IBA-1 expression in adulthood [67,68].

Our group (2018) demonstrated that a milder variant of the social defeat paradigm, when applied during the periadolescent period, is able to increase microglia number, IBA-1 expression, and soma size in the ventral tegmental area of pups. In adulthood, cell density returned to control levels, but microglia displayed increased soma size and process complexity close to the soma. Interestingly, the microglial cells of stressed mice showed a higher IBA-1 percentage of expression in response to cocaine injection (5 mg/kg) with respect to controls, indicating a possible effect of early social stress on microglial priming [69]. Differently, a paradigm of *social instability stress* applied during adolescence (isolation and change of cage partner) did not produce effects on counts of microglia in the dentate gyrus (HIP) either early or later after the stress exposure [70].

In order to evaluate how combined stressors during postnatal development affect microglia, MS has been followed by *adolescence stress* (AS) paradigms. In these studies, AS and AS paired with MS had a higher impact on microglia than MS alone. Han et al. (2019) observed that HIP microglia from MS mice tended toward a more hypertrophic phenotype and a lower CX3CR1 expression with respect to controls, and this effect became prominent after exposure to adolescent restraint stress [71]. These mice showed an increase in pro-inflammatory markers (inducible nitric oxide synthase, iNOS, tumor necrosis factor α, TNF-α, interferon gamma, IFN-γ, IL-6, and IL-1β) and downregulation of several anti-inflammatory markers (IL-4, transforming growth factor β, TGF-β, IL-1 receptor agonist, IL-1Rα, Ym-1, Arginase 1, Arg1) in the HIP, which were rescued by minocycline treatment. In another study with adolescent female rats, MS alone did not alter microglial number, but induced an activated phenotype in the PFC and HIP. In contrast, adolescent mild, variable stress increased microglial cell count, an effect that was further amplified in rats who underwent both the stressful procedures [72]. Finally, combining MS with adolescent food restriction was able to increase IBA-1 expression in the PFC of both sexes compared to only MS animals [73] (see Table 2 for a summary).

#### 2.1.5. Postnatal Environmental Agents and Infection

A large body of literature focusing on *alcohol* exposure in early postnatal life describes an increase in microglial activation markers and phenotype changes in the cortical and subcortical regions of the brain immediately after treatment [77,78,79,80,81,82,83,84,85,86]. Regarding cell density, contrasting findings have been collected, with some studies showing an increase [78,86] and others a decrease [79,85,87] in the number of microglial cells in both cortical and subcortical compartments. It has also been shown that alcohol induces an increase in amoeboid microglia along with a decrease in resting microglia in the HIP and cerebellum [82]. Moreover, Ahlers and colleagues (2015) observed layer-specific differences of microglial responses to ethanol in the somatosensory cortex (increase/decrease in number, alteration of CD68 expression, and different morphologies) [88]. Notably, one study reported no acute or late effect of early postnatal alcohol exposure on microglial markers in the visual cortex [89].

Interestingly, adolescent *binge drinking* seems to have long-lasting effects on microglia [83,90], whereas the temporal extent of microglial activation in neonates exposed to alcohol is still not clear. Topper and colleagues reported that alcohol inhalation on P3–5 has transient effects on HIP and cerebellar microglial activation [82]. However, Chastain and colleagues (2019) observed an increase in microglia cell count in the hypothalamus of alcohol-fed pups (P2–6) that persisted into adulthood. At P6, this effect was associated with altered microglial gene expression, including augmented transcripts of IL-6, TNF-α, CSF1R, and TLR4. In adulthood, similar molecular alterations were detected only in alcohol-fed rats that received an LPS immune challenge later in life. These LPS-induced effects were rescued in animals administered minocycline during alcohol feeding, suggesting a priming effect of early ethanol on microglia [78].

Two studies identified contradictory results regarding sex differences in the microglial responses to early *alcohol exposure*. There is evidence of a female-specific increase in the number of HIP microglial cells in young (P5) rats acutely exposed to ethanol [86]. However, in mice, alcohol exposure had the same effects on microglia in both sexes [88].

Among opioid drugs, morphine has been shown to increase microglial TLR4 expression in the nucleus accumbens (not in the HIP), when administered in adolescence, but not adulthood. Adult morphine re-treatment increased CD11b expression solely in pre-exposed rats (priming effect) [91].

Early-life exposure to high doses of potentially toxic chemicals (GBR12909 [92], valproate [93], methylphenidate [94], manganese [95]) impacts microglia with very similar results in the short term. Specifically, increases in cell number, amoeboid/hypertrophic morphology, and expression of activation markers (CD68) have been found across brain regions, including frontal and other cortices, HIP, AMY, striatum, thalamus, substantia nigra, and globus pallidus [92,93,94,95,96].

However, discrepant results have been reported on the long-term effects, wherein studies demonstrated either a persistent increase in microglial activity (GBR12909 [92]) or no effect (manganese [95]).

Bacterial infection is one of the most common immune challenges occurring in early life. Exposure to LPS in the first postnatal week produces a rise in microglial cell density that lasts for a few days after the injection [97,98,99]. Pang and colleagues (2016) reported an acute LPS-induced increase in the number of microglial cells, which mostly colocalized with markers of an anti-inflammatory profile (TGF-β, CD206), except for few pro-inflammatory-profiled cells (expressing CD68, major histocompatibility complex-II, iNOS) [99]. Claypoole et al. (2017) found sex differences in this response, with increase found only in female HIP [97]. Notably, the timing of LPS exposure is determinant for microglial responses, given that injection at P2, but not at P21, results in an immediate increase in microglial activation markers (CD11a, F4/80, CD172a, SLAM family member 7) [100]. The authors suggested that this increased neuroinflammatory response to bacterial molecules in neonatal but not periadolescent pups may underlie the sensitivity to infection that characterizes early postnatal neurodevelopment. Additionally, early LPS administration has been shown to have a long-lasting effect on microglial responses: injection applied during the first or second postanal week increases microglial cell density, IBA-1 expression, and hypertrophic morphology later in life, registered in the PFC [101,102], HIP [101,103,104], and substantia nigra [105,106,107]. Moreover, high colocalization between apoptosis-associated speck-like protein (ASC, part of the NLRP3 inflammasome) and IBA-1 was found in adolescent brains from LPS-exposed rats [101], suggesting an effect on apoptotic pathways in microglia.

Seminal works from Bilbo and colleagues shed light on numerous effects of early postnatal immune activation on microglial function and related behavior. Using a model of neonatal infection (NI) through *Escherichia coli* (*E. coli*, a Gram-negative bacterium) exposure at P4, they reported a marked increase in microglial activation markers (CD11b mRNA) within the HIP of NI rats early after the infection. This increase persisted into adulthood and was exaggerated by a second challenge with LPS [108,109]. Moreover, the HIP from NI pups exhibited a higher count of microglial cells, which were shown to be active and proliferating [110], but this number returned to control levels in adolescence. However, in adult NI mice, microglia exhibited an altered profile, with larger cell volumes; shorter, thicker processes; and sustained increase in the expression of activation markers [107,108,109]. These long-term alterations are associated with sensitized microglia and, indeed, microglia from NI mice show exaggerated reactivity to various types of stimuli that function as a second challenge later in life, from LPS [108,109] to amphetamine [111] and fearful stimuli [112].

#### 2.1.6. Effects of Postnatal-Stress-Induced Microglial Alterations on Brain and Behavior

In the context of ELS, it is crucial to understand the possible mechanisms by which altered microglial activity can, in its turn, influence neuronal function and behavior. However, the literature still lacks an integrative understanding of microglial responses to postnatal ELS and their effects on brain and behavior development. Very few studies have analyzed the impact of MS on microglial–neuronal interaction, showing region-specific effects in the CNS. For example, Han and colleagues (2019) investigated the molecular profiles of HIP microglia in mice exposed to MS, showing decreased expression of genes involved in microglial–neuronal communication, such as CX3CR1, immediately after the stress procedure. This effect was exacerbated by exposure to adolescent *restraint stress* [71]. In contrast, no changes in expression of the CX3CL1/CX3CR1 pathway were observed in medullar microglia from MS mouse pups [60]. A combination of MS with *neonatal handling*, which increased maternal care towards the pups, induced a decrease in CX3CL1 expression in the nucleus accumbens (but not the HIP) of adult rats. Interestingly, microglial activation in this structure was associated with behavioral alterations consisting of attenuation of morphine-induced reinstatement of conditioned place preference [75].

Several studies have implicated microglia in the process that links AS to the brain and behavioral alterations later in life. In a model of *adolescent sleep deprivation*, decreases in CX3CR1, CD11b, and P2Y12 were observed in the HIP of young mice, with no alteration in CSF1R levels or microglial cell density. In parallel, an increased density of excitatory synapses and reduced CD68 expression was observed. This was associated with reduced engulfment of postsynaptic density by microglia in adolescence but not in adulthood [76]. *Chronic social isolation* starting at weaning induced an increase in the expression of IBA-1 and CD11b and a decrease in CD200R mRNA in the HIP of rats, along with neuronal epigenetic alterations that were rescued by poststress minocycline treatment [74]. Moreover, minocycline could inhibit the reduction in proliferative neuronal cells, and the downregulation of BDNF was detected in the HIP of juvenile mice that underwent combined MS and AS procedures [71]. In parallel, this treatment could also rescue stress-induced depressive-like (but not anxiety-like) behavior. Similarly, minocycline decreased the levels of depressive-like behavior in socially isolated rodents [66,74]. Minocycline treatment during *adolescent social defeat* rescued cognitive deficits observed in adulthood [67]. Recently, our group (2018) demonstrated that *periadolescent social stress*-induced microglial activation mediated dopaminergic system dysfunctionality and cocaine-induced behavioral alterations. Indeed, these effects were rescued by either minocycline or GW2580 (selective inhibitor of CSF1R) administration during the stress procedure [69].

Regarding other harmful forms of ELS, Ahlers and colleagues (2015) reported an increase in the contacts between microglia and apoptotic pyramidal cells in the somatosensory cortex after exposure to early postnatal ethanol, suggesting the engulfment of neuronal residues by microglia. Moreover, they demonstrated that microglial activation was driven by alcohol-induced apoptotic cell death, and not by the direct effects of alcohol on microglia [88]. In an experiment of neonatal immune activation, minocycline pretreatment before LPS injection failed to rescue the locomotor, anxiety, and depression-like behavioral alterations in adult mice [102]. However, a more extended treatment (minocycline exposure pre- and post LPS injection) significantly attenuated LPS-induced brain injury and improved neurobehavioral performance in P21 rats [106]. Bilbo and colleagues extensively characterized the molecular profiles of sensitized microglia in the *E. coli* model of NI. A region-specific modulation of the CD200/CD200R and CX3CL1/CX3CR1 pathways in adult NI rats was observed in nucleus accumbens and HIP microglia cells following a second challenge with amphetamine or a fearful stimulus later in life [111,112]. Interestingly, animals exposed to NI and LPS showed memory impairments in a fear conditioning protocol, which were prevented by minocycline treatment before the LPS.

Drugs of abuse such as morphine can sensitize microglia when administered in early life. Indeed, inhibiting microglial activation through ibudilast (but not minocycline) co-injection during adolescence attenuates morphine-induced glial activation and behavioral responses in adult rats [91]. (see Table 3 for a summary)

#### 2.1.7. Early Preventative Interventions

As a result of the growing body of evidence suggesting the role of microglia in mediating the effects of ELS on psychopathological behavior, it has become essential to develop and test potential interventions that can directly target these cells [113]. In this section, we discuss several preclinical rodent studies that explored the capability of different pharmacological and environmental manipulations of mitigating microglial activation induced by exposure to ELS.

*Environmental enrichment* (EE) has been widely investigated for its efficacy in preventing the effects of ELS on maladaptive behavioral phenotypes in adulthood [114,115,116,117]. The mechanism by which EE exerts this effect is poorly understood. Preclinical evidence suggests that EE may ameliorate the effects of ELS by interacting with the immune system and preventing elevations in circulating pro-inflammatory cytokines TNF-α, IL-1β, and IL-10 [118,119]. While there is limited evidence linking ELS, EE, and microglia dysregulation, EE has been shown to reduce microglial proliferation compared with standard housing in brain regions such as the AMY and the HIP and to modulate microglia density in the cerebral cortex [120,121]. Interestingly, EE failed to prevent long-term alterations to microglial density due to prenatal LPS injections [122], but prevented the elevation of pro-inflammatory cytokines and phagocytic activity when LPS was administered during adolescence and adulthood in the HIP [123,124]. This evidence suggests that EE may only be useful as a treatment model for certain ELS at specific developmental periods.

Oxytocin (OXT) is a peptide hormone that plays a significant role in social bonding, sexual reproduction, childbirth, and early development. As a result of ELS, OXT pathways in the hypothalamus, AMY, and PFC are disrupted [125,126]. While there is little evidence identifying how the administration of OXT affects behavior after ELS, there is a growing body of literature suggesting that it may serve as a useful target for both pharmacotherapy and prevention. Yuan et al. (2016) demonstrated that intranasal administration of OXT to adult mice reduced microglial activation and pro-inflammatory cytokine expression induced by an LPS injection in the PFC of adult mice. In addition, the authors demonstrated that OXT could reduce LPS-induced activation both in primary microglia and in microglial cell lines [127]. Recently, Amini-Khoei et al. (2017) demonstrated that intracerebral administration of OXT reduced pro-inflammatory gene expression related to microglia activity, including TNF-α, IL-1β, and TLR4, in the HIP of adult animals exposed to MS [128]. In a double-hit model using a low-protein diet to induce perinatal brain damage followed by postnatal injections of IL-1β during early life, the accompanying administration of the OXT receptor agonist carbetocin was found to prevent microglial activation and reduce the effects of the low-protein diet and IL-1β injections on genes associated with microglial activation throughout the brain [45].

Galantamine is a tertiary alkaloid that acts as a reversible and competitive inhibitor of the alpha 7-nicotinic acetylcholine receptors (α7-nAChrRs) [129]. Through this receptor subunit on microglia, galantamine has been demonstrated to modulate microglia activation [130,131]. While galantamine has been most widely explored as a treatment for Alzheimer’s disease [132], there is growing evidence that it can help to regulate microglia and ameliorate the behavioral effects related to ELS [131,133,134,135,136,137]. Oral administration of galantamine during adolescence reversed memory deficits in adulthood caused by maternal separation [134] and ameliorated the effects of perinatal exposure to kynurenic acid on cognitive flexibility in a mouse model of schizophrenia [137].

Melatonin is a hormone that is mainly secreted through the pineal gland [138]. Preclinical work suggests that melatonin can reduce microglial alterations in brain regions such as the AMY, hypothalamus, ipsilateral basal cortex, and HIP [139,140,141]. Melatonin can modulate microglia density and morphology as well as phagocytic response to LPS in the HIP both in adulthood [142,143] and in early life [102]. Furthermore, melatonin treatment during gestation has attenuated microglia activation in the HIP as a result of umbilical cord occlusion in early life, and kainic acid administration via intraperitoneal injection in adulthood [144,145].

Long-chain polyunsaturated fatty acids such as omega-3 (ω-3) have been widely investigated for their ability to alter behavior and the immune system [146]. ω-3 fatty acids can reduce microglial activation and the release of pro-inflammatory cytokines [146,147,148,149,150]. In addition to their effects on the immune system, supplementation of ω-3 fatty acids in adulthood can reduce anxiety-like and depression-like behaviors following adolescent LPS injections [151]. Consistent with the behavioral outcomes found in adulthood, early-life supplementation of ω-3 has been shown to prevent increases in anxiety-like and depression-like behavior following ELS [152,153]. Moreover, ω-3 fatty acid supplementation during gestation and early life has been shown to prevent alterations to the development of microglia in the HIP, while also reducing cognitive deficits following ELS [55,154].

### 2.2. Early-Life Stress and Microglia: Clinical Studies

Although the impact of ELS on microglia has been primarily investigated in a preclinical context, this aspect has rarely been studied in humans. The reason for this lies in the severe methodological limitations to the study of microglial morphology and functionality in the brains of living humans. Moreover, human postmortem brain studies have never investigated the long-term impact of ELS on microglia. Insights on this topic were offered by a recent PET study that used TSPO radioligand administration. In this study, PET scans were used to assay the relationship between childhood traumatic (abuse/neglect) experiences and central TSPO radioligand binding. The authors failed to identify significant differences in TSPO expression in the frontal, parietal, temporal, and occipital lobes when comparing healthy individuals with or without a history of exposure to psychosocial risk early in life [155]. This suggested that the exposure to ELS in humans did not induce a stable microglial activation. While this evidence contradicts the preclinical results, it could be due to several possible reasons. For example, in this study, only ELS individuals without a psychiatric diagnosis were recruited, suggesting that primarily “resilient” individuals were represented in the study, while the “ELS-vulnerable” population, with potentially more sensitive microglia, were excluded.

While TSPO radioligand administration is the only procedure that has been used to investigate the impact of ELS on microglia, other targets for PET studies may prove to be more valuable. For example, Horti et al. (2019) recently developed [11C]CPPC, a positron-emitting, high-affinity ligand that is specific to macrophage CSF1R, which is primarily restricted to microglia in the brain. [11C]CPPC demonstrates high and specific brain uptake in a murine and nonhuman primate LPS model of neuroinflammation. It also shows specific and elevated uptake in a murine model of Alzheimer’s disease and postmortem brain tissue of patients with the disease, suggesting that [11C]CPPC may represent a promising tool to target microglia in the human brain [156]. Although researchers are currently developing techniques to investigate microglial activity in humans, much more work is required to identify potential targets for future research and test whether existing methods are valid and reproducible.

In addition to the fact that there have been no studies to date identifying the role of microglia following ELS in clinical populations, there is no literature investigating the potential benefits of early interventions following ELS. There are several studies, however, that demonstrate the involvement of both ω-3 fatty acids and oxytocin in development. For example, higher levels of ω-3 fatty acids in maternal milk are associated with better temperament in children [157], and reduce negative affect and inflammation in children that have mothers with high levels of adiposity [158], while lower levels of oxytocin have been related to experiences of severe emotional neglect and abuse in childhood in plasma and cerebrospinal fluid samples [159,160]. Children raised in orphanages who experienced neglect have also been shown to have lower oxytocin plasma levels [161]. Additionally, mothers who reported experiencing childhood trauma had lower oxytocin plasma levels and engaged in less positive parenting styles [162]. Despite this evidence, it is not known if microglia are involved in these ω-3 fatty acid and oxytocin effects on children and whether this involvement is also observable in the presence of ELS.

### 2.3. Perspectives

The literature collected and described in this review shows that ELS has robust effects on microglial functionality and morphology, and that ELS-induced microglial activation mediates the emergence of brain and behavioral alterations. Interestingly, these effects strongly depend on the developmental timing of ELS application. This may be due to a diverse sensitivity of microglial and neuronal processes across development [2,163]. Indeed, during development, microglia undergo different stages of maturation (from brain colonization to acquisition of an adult phenotype) associated with specific functional roles [164]. In this review, we showed that ELS interferes with microglial developmental programs, including their proliferation and death as well as their phagocytic activity. This, in turn, can affect neuronal and non-neuronal developmental processes, which are dynamic during development [68,74] and for which microglial activity is instrumental. The effects of ELS on microglia also depend on the time point of investigation. This dependency may result from changes in local environmental cues associated with acute or delayed effects of the stress [165]. Indeed, microglia reactivity is determined by these cues [164,166]. Furthermore, we have presented evidence for a priming effect of both pre- and postnatal ELS on microglial reactivity, indicating a long-lasting alteration, which has been implicated in behavioral abnormalities later in life. In conjunction with these findings, sex-related differences in microglial responses to ELS and consequent behavioral outcomes have been found. However, studies using different ELS procedures have yielded different outcomes. As previously described by Bordt, Caesrine, and Bilbo (2019), sex differences in microglial number and morphology exist during critical periods of development [167]. These developmental differences may mediate the diverse sex-specific reactivity to ELS [168]. Further research is required to determine how these varying ELS models facilitate these differences.

Although the literature surrounding ELS and microglia has grown exponentially, there are several limitations within the existing body of work that still need to be addressed. First, many of these studies have focused on one or few target structures for investigation, particularly the HIP. As reviewed by Tan, Yuan, and Tian (2019), microglia show regional heterogeneity in density, morphology, and phenotype, which may underlie differential responsivity to stimuli, including stress and inflammatory conditions [69,169]. This spatial heterogeneity could be important for tuning neuronal/glial functions and neural circuitry, thus contributing to the region-specific effects of ELS on the brain. Recently, deep single-cell RNA sequencing (scRNAseq) data from isolated microglia cells has revealed several transcriptionally distinct clusters throughout each developmental stage [170,171,172]. Based on this evidence, Stratoulias and colleagues (2019) hypothesized that microglia constitute a heterogeneous population, in which each cell displays intrinsic properties and functional specializations regardless of the structure [173]. As a result, neighboring microglial cells may have different responses to the same stimulus. Therefore, further research is necessary to investigate how microglial subsets act in an ELS context. In addition, microglia have been analyzed phenotypically and functionally using cellular markers that are also expressed on central-nervous system-associated macrophages and peripheral innate immune cells. This is relevant in the context of ELS, due to the fact that ELS has been shown to induce BBB leakage [174]. With this increase in permeability, peripheral immune cells have been proposed to enter the brain [175]. Notably, these populations may bolster microglial diversity, as observed by a recent transcriptome study by Prinz and colleagues [176]. While the role of these immune cells in brain development following ELS has yet to be explored, it is possible that some of the outcomes associated with ELS may be facilitated via the actions of the peripheral immune system [177]. Moreover, rodent experiments have shown that peripheral inflammatory stimuli can affect microglia in the brain [178]. Using markers that target subpopulations of microglia and macrophages such as transmembrane protein 119 (TMEM119), P2Y purinoceptor 12 (P2RY12), and Sal-like protein 1 (SALL1) [10,179], it would be valuable to identify how various types of microglia and resident/peripheral macrophages relate to alterations in brain development and behavior following ELS.

There is still a gap of knowledge surrounding the mechanisms through which ELS impacts microglia, and how microglial modifications mediate ELS health-related outcomes. Both genetic and epigenetic factors are thought to play a role in the long-term effects of ELS [180]. Currently, no study has investigated the contribution of microglia to these factors in the context of ELS. Future bioinformatic analyses should explore whether genetic risk factors and/or epigenetic modifications associated with ELS health outcomes are related to microglia function and activity [181].

While research continues to implicate microglia as mediators in a host of psychopathologies, identifying preventive strategies to reduce the impact of ELS on microglia is of the utmost importance. In this review, we have presented several new measures that may prevent ELS impact on microglia. While these models show promise, further research is required to elucidate whether and how the considered measures can be used to prevent the effects of ELS on microglia and, consequently, on behavioral outcomes, particularly in the clinical setting.

## 3. Conclusions

Overall, preclinical research supports the hypothesis that ELS can exert its effects on brain and behavior through microglia-mediated processes. This evidence should be used to drive clinical research to verify and confirm the relevance of this phenomenon in humans. However, several limitations delay this research, including (1) the paucity of suitable radioligands for studying microglia by PET; (2) the difficulty of identifying the effects of specific ELS events on the brain, due to the fact that multiple stressful stimuli often co-occur during development, which distorts the original cause of an alteration; (3) evaluating the impact of pre- and postnatal stressful events on brain and behavior requires extensive and longitudinal studies where subjects should be monitored in terms of mental and physical health throughout development and into adulthood. Future efforts are required to investigate microglia in humans and, using novel techniques, understand how microglia relate to ELS and psychopathology.

## Figures and Tables

**Table 1:** Effects of prenatal ELS on microglial cell density and phenotype and subsequent behavioral alterations.

	Type of Stress	Time	Species and Sex	Early Effects on Microglia	Late Effects on Microglia	Brain Region	Microglial-Dependent Behavioral Effects	Ref
**Behavioral stressors**	Maternal forced swim	E18–P21	Rats, M F	P1: ↓lectin+cells and amoeboid morphology (in CC); ↑ lectin+ cells (other regions)	-	CC, PCtx, ECtx, PFC, Sp, BG, Th, Md, IC	-	[28]
	Maternal sleep deprivation	E18–P1	Rats, M	-	P21: ↑IBA-1+ cells and retracted thicker processes	HIP	↓ spatial learning & memory	[29,30]
	Maternal restraint stress/LPS	E12–P21/P120	Mouse, F	P120: ↑ IBA-1+ cells, ↑ soma size and thick processes/↑ IBA-1+ cells	-	HIP (CA1/DG)	-	[31]
**Environmental agents and infection**	Maternal Poly I:C injection	E15	Rat, M	-	P103: ↑ microglial IL-1β, TNFα	HIP	↑ startle response in the PPI	[34]
		E9	Mouse, M F	-	P62: ↑ number, amoeboid IBA-1+ cells	Ctx, HIP, Th	↑locomotor activity and startle response in the PPI, ↓ sociability	[36]
		E9.5	Mouse, M F	-	P90: ↑ IBA-1+ cells, ↓ arborization in M	PFC, HIP, Cb	-	[39]
		E14.5	Mouse, M F	P1: adult-like transcriptome	P28: no effect	WB	-	[49]
		E15	Mouse, M		P80: ↑IBA-1+ cells, ↓phagocytosis	HIP (DG)	↑ startle response in the PPI, ↓working memory in novel object recognition	[50]
		E15	Rat, M F	-	P180:↑ OX-42+ cells (in CC), activated, ↓microglia arborization (in HIP)	CC, HIP	-	[35]
	Maternal Poly I:C injection/unpredictable stress	E9/P30–40	Mouse, M F	P41: ↑ CD68+ cells, ↑ soma size	-	PFC, HIP	↑ startle response in the PPI, anxiety-like behavior, amphetamine-induced locomotion	[37]
	Maternal LPS injection	E9	Mouse, M F	-	P15: ↓ CX3CR1 mRNA in M	HIP	-	[38]
		E13.5	Mouse, M F	-	P7: = number of IBA-1+ cells	SCtx	-	[40]
	Maternal high fat diet/limited nesting	E13–P21	Mouse, M F	P21: ↑ CD11b+ and IBA-1+ cells	-	HIP	-	[43]
	Maternal high fat diet	E0–E17	Mouse, M F	E17 (LPS in vitro): ↑ TNF-α release in M	-	WB, Plac	-	[44]
	Maternal low protein diet/IL-1β	E10–P1/P1-2	Rat, M F	P4: ↑ microglial inflammatory genes; ↑ IBA-1+ amoeboid cells (in vitro)	---	WB, Ctx WM	-	[45]
	Maternal alcohol exposure	E6–18	Mouse, M F	E15.5: ↑ IBA-1+ amoeboid cells	P3:↑ IBA-1+ amoeboid cells, ↓ ramified	PFC	-	[46]
	Diesel exhaust particles exposure/high fat diet	E2,5,8,12,16 /P120–183	Mouse, M F	P183: ↑ CD11b and CX3CR1 mRNA in M	-	HIP	-	[47]
	Diesel exhaust particles exposure	E2,5,8,12,16	Mouse, M F	-	P30: = number of IBA-1+ cells, ↑ cell volume, microglia-neuron interaction	PCtx	-	[48]

Abbreviations: BG: basal ganglia; CA1: cornu ammonis 1; Cb: cerebellum; CC: corpus callosum; CD clusters of differentiation; Ctx: cortex; DG: dentate gyrus; E: embryonic day; ECtx: entorhinal cortex; F: female; HIP: hippocampus; IBA-1: ionized calcium-binding adapter molecule; IC: internal capsule; IL: interleukin; LPS: lipopolysaccharides; M: male; Md: medulla; P: postnatal day; PCtx: parietal cortex; PFC: prefrontal cortex; Plac: placenta; PPI: prepulse inhibition; SCtx: somatosensory cortex; Sp: septum; Th: thalamus; WB: whole brain; WM: white matter.

**Table 2:** Effects of postnatal behavioral stressors on microglial cell density and phenotype and subsequent behavioral alterations.

	Type of Stress	Time	Species and Sex	Early Effects on Microglia	Late Effects on Microglia	Brain Region	Microglial-Dependent Behavioral Effects	Ref
**Behavioral stressors**	Glucocorticoids exposure	P3–5	Rat, M F	-	P12: ↓ number (Hortega method)	WM	-	[51]
		P0	Rat, M F	P2, 5: ↓ amoeboid microglia (Hortega method)	P10: ↑ branching	CC	-	[52]
		P1,3,5	Rat, M F	P4,7: ↓ amoeboid OX-42+ cells	P8: ↑ branching	CC	-	[53,54]
	Limited nesting	P2–9	Mouse, M	-	P245: ↑ CD68+ cells	HIP	Cognitive deficits	[55]
		P2–9	Mouse, M	P9: ↓ IBA-1+ cells/processes complexity	-	ECtx/HIP	-	[56]
	Maternal separation	P1-14	Rat, M	P15: ↑ activated IBA-1+ cells	-	HIP (Hilus)	-	[57]
		P1–21	Mouse, M	P14: ↑ number, activated IBA-1+ cells; P14, 28: transcriptomic alterations	-	HIP	-	[58]
		P1–14	Rat, M	P15: ↓ number, ↑ activated IBA-1+ cells	-	HIP (Hilus, CA3)	-	[59]
		P3–12	Rat, M F	P 14: ↑ number, soma size, ↓ arborization area of IBA-1+ cells	-	Md	-	[60]
		P1–10	Rat, M	P 10: ↑ IBA-1 IR	P20, 30, 60: ↑ IBA-1 IR and mRNA; P40,50: no effect	HIP, PFC	-	[61]
		P2–14	Rat, M F	P15: ↓ in CD11b+ apoptotic cells	-	VTA, SN (only M)	-	[62]
		P1–21	Rat, M	-	P100: ↑ IBA-1+ cells	DS, NAc, HIP (CA3)	-	[63]
		P2–12	Rat, M F	-	P84: ↓ IBA-1 mRNA	SC	-	[64]
		P2–14	Mouse, M	-	P60: ↑ motility of IBA-1+ cells	SCtx	-	[65]
	Social isolation	P14–21	Mouse, M F	-	P70: ↓ number, soma size and processes of IBA-1+ cells	HIP	↑ depressive-like behavior	[66]
		P21–63	Rat, M	P63: ↑IBA-1 IR, CD11b, ↓ CD200R mRNA	-	HIP	↑ depressive-like behavior	[74]
	Social defeat	P28–37	Mouse, M	P28/38:↑ IBA-1 IR	P80: ↓ IBA-1 IR and IBA-1+ cells	PFC	Cognitive deficits	[67,68]
	Social stress	P14–21	Mouse, M F	P22: ↑ IBA-1 IR and IBA-1+ cell number, soma size	P60: ↓ IBA-1 IR, ↑ soma size and processes complexity	VTA	↑ cocaine CPP	[69]
	Social instability stress	P30–45	Rats, M	P33,46: no effects on OX-42+ cells number	P75: no effects	HIP (DG)	-	[70]
	Maternal separation/restraint stress	P1–14/P42–56	Mouse, M	P42: ↑ activated IBA-1+ cells, ↓ CX3CR1 mRNA/P56: ↑ pro-infl, ↓ anti-infl cytokines mRNA and IR	-	HIP	↑ depressive-like and anxiety-like behavior	[71]
	Maternal separation/mild variable stress	P2–21/22–42	Rat, F	P51: ↑ activated IBA-1+ cells and IBA-1 IR	-	PFC, HIP	-	[72]
	Maternal separation+food restriction/food restriction	P2–20+P36–55/P36-55	Rat, M F	P55: ↑ IBA-1 IR both sexes/↑ IBA-1 IR and ramification only in F	-	PFC	-	[73]
	Maternal separation+handling	P2–20	Rat, M F	-	P60: ↓ CX3CL1 mRNA	NAc, not HIP	↓ morphine CPP and reinstatement	[75]
	Sleep deprivation	P35	Mouse, M	P38: ↓ CD68 IR, CX3CR1, CD11b and P2Y12 mRNA, ↓ ramification and PSD95 engulfment, no effect on number and CSF1R mRNA	-	HIP	-	[76]

Abbreviations: CA3: cornu ammonis 3; CC: corpus callosum; CD clusters of differentiation; CPP: conditioned place preference; Ctx: cortex; DG: dentate gyrus; DS: dorsal striatum; ECtx: entorhinal cortex; F: female; HIP: hippocampus; IBA-1: ionized calcium-binding adapter molecule; IR: immunoreactivity; M: male; Md: medulla; NAc: nucleus accumbens; P: postnatal day; PFC: prefrontal cortex; SC: spinal cord; SCtx: somatosensory cortex; SN: substantia nigra; VTA: ventral tegmental area; WM: white matter.

**Table 3:** Effects of postnatal environmental agents and infection on microglial cell density and phenotype and subsequent behavioral alterations.

	Type of Stress	Time	Species and Sex	Early Effects on Microglia	Late Effects on Microglia	Brain Region	Microglial-Dependent Behavioral Effects	Ref
Environmental agents and infection	Alcohol exposure	P7	Rat, M	P8: ↑ IBA-1 IR	-	Ctx	-	[77]
		P2–6	Rat, M F	P6: ↑CD11b+ cells, IL-6, TNF-α, CSF1R, TLR4 mRNA and epigenetic alterations	P90:↑ IBA-1+ and CD11b+ cells	HYP	-	[78]
		P4–9	Rat, M	P10: ↓ ramification of IBA-1+ cells	-	HIP	-	[79]
		P35–39	Rat, M F	P40: ↑ activated IBA-1+ cells	-	HIP	-	[80]
		P50–71	Rat, F	P71: ↑ activated OX-6 (CD74)+ cells	-	HIP	-	[81]
		P3–5	Rat, M	P6: ↑ amoeboid and ↓ resting IBA-1+ cells	-	HIP, Cb	-	[82]
		P35–38	Rat, M	P40: ↑ activated IBA-1+ cells, BrdU+	P65: ↑ activated IBA-1+ cells	HIP	-	[83]
		P4–9	Mouse, M F	P10: ↑ IBA-1 IR, ↓ processes	-	PCtx, HIP, Cb	-	[84]
		P3–5	Mouse, M F	P6: ↓ isolectin+ cells	-	Cb	-	[85]
		P4	Rat, M	P5: ↑ CD11b in F, not M	-	HIP	-	[86]
		P35–90	Rat, M	P90: ↓ IBA-1+ cells	-	MCtx	-	[87]
		P7	Mouse, M F	P8: ↑ amoeboid CX3CR1-GFP/+ cells and CD68 IR; P9: return to control levels	-	SCtx	-	[88]
		P4-9	Mouse, M F	P5/10: no effects	P28: no effects	VCtx	-	[89]
		P25–55	Rat, M	-	P80: ↑ in IBA-1+/NF-κB+ cells	HIP	-	[90]
	Morphine exposure	P37–42	Rat, M	P43: ↑ microglial TLR4 mRNA and IR	P60: ↑ CD11b mRNA after morphine re-exposure	NAc	Reinstatement of morphine CPP	[91]
	Oxidative Insult (GBR12909)	P10-20	Mouse, M F	-	P60:↑IBA-1+ and CD68+ cells	HIP	-	[92]
	Valproate exposure	P7	Rat, M	P8: ↑ IBA-1+ cells, ↑ ramification	-	VCtx, HIP, AMY	↑ depressive-like and anxiety-like behavior	[93]
	Methylphenidate exposure	P28–55	Rats, M F	P57: ↓ ramification of IBA-1+ cells, ↓ CX3CR1 mRNA	-	PFC	-	[94]
	Manganese exposure	P20–34,90–150	Mouse, M F	P35: ↑ameboid IBA-1+ cells, NOS2+; P150: no effect	-	DS, NAc, SN, GP	-	[95]
	Methylphenidate exposure	P21–111	Rat, M	P112: ↑ [3H] PK 11195 binding	-	Ctx, Th, GP, SN	-	[96]
Environmental agents and infection	LPS injection	P3–5	Rat, M F	P7: ↑ IBA-1+ cells in F	-	HIP		[97]
		P4	Mouse, M F	P7-10: ↑IBA-1 mRNA and IBA-1+ cells	-	Ret	-	[98]
		P3	Rat, M F	P6: ↑ IBA-1+ cells, amoeboid-like/rod soma, ↓ ED1, MHC-II, iNOS+ cells, ↑ TGF-β, CD206+ cells	P21: = number, ↑ soma size and ↓ processes	HIP, WM, PV	-	[99]
		P2,21	Mouse, M F	P21 (vs P2): ↑ microglial CD11a, CD172a IR, ↓ SLAMF7 IR	-	WB	-	[100]
		P14,15	Rat, M F	-	P35: ↑IBA-1+ hypertrophic, apoptotic cells	PFC, HIP	-	[101]
		P6	Rat, M	-	P90: ↑ IBA-1+ cells	PFC	-	[102]
		P3,5	Rat, M	-	P85: ↑IBA-1 soma expression	HIP (CA1, DG)	-	[103]
		P5	Rat, F	-	P71: ↑ OX-42+ cells	HIP (CA1)	-	[104]
		P5	Rat, M	-	P70: ↑ OX-42+ cells	SN	-	[105]
		P5	Rat, M	-	P21: ↑ activated OX-42+ cells	CC, PV	↓ neurobehavioral performance	[106]
		P4–6	Mouse, M F	P7: ↑ proportion of amoeboid cells, P11: ↓ CX3CR1-GFP+ cells	P13-15: ↑ CX3CR1-GFP+ cells, ↑ proportion of amoeboid cells	Pons	-	[107]
	E.Coli/LPS injection	P4/P60	Rat, M	P5,6,7: ↑ CD11b expression	P60: ↑ CD11b mRNA, further ↑ by LPS	HIP	-	[108]
		P4/P60	Rat, M	P6: ↑ number, active and proliferating IBA-1+ cells	P33: no changes/P60: ↑ IBA-1+ cells number and volumes, further ↑ by LPS	HIP, PCtx (not FC)	-	[110]
	E.Coli/Handling/LPS	P4/P4–20/P60	Rat, M	-	P60: ↑ CD11b mRNA, further ↑ by LPS, ↓ by handling	HIP	-	[109]
	E.Coli/Amphetamine	P4/P40	Rat, M	-	P40: ↑ CD200 mRNA by amph, ↓ in E.Coli-amph	NAc	-	[111]
	E.Coli/LPS+Fear conditioning	P4/90	Rat, M	-	P90: ↑ CD11b,↓ CD200, CD200R, CX3CR1 mRNA, ↑ microglial IL-1β mRNA (further ↑ by LPS)	HIP	↓ memory performance	[112]

Abbreviations: AMY: amygdala; CA1: cornu ammonis 1; Cb: cerebellum; CC: corpus callosum; CD clusters of differentiation; CPP: conditioned place preference; Ctx: cortex; DG: dentate gyrus; DS: dorsal striatum; F: female; GP: globus pallidus; HIP: hippocampus; HYP: hypothalamus; IBA-1: ionized calcium-binding adapter molecule; IL: interleukin; IR: immunoreactivity; LPS: lipopolysaccharides; M: male; MCtx: motor cortex; NAc: nucleus accumbens; P: postnatal day; PCtx: parietal cortex; PFC: prefrontal cortex; PV: periventricular areas; Ret: retina; SCtx: somatosensory cortex; SN: substantia nigra; Th: thalamus; VCtx: Visual cortex; WB: whole brain; WM: white matter.
